# Effects of Rice–Duck–Crayfish Integrated System on the Community Structure of Plankton and Its Relationships with Environmental Factors

**DOI:** 10.3390/biology15060501

**Published:** 2026-03-20

**Authors:** Yuchen Jing, Zhiwei Xu, Mengmeng Pan, Jiaqian Yu, Zehua Fang, Xufa Ma, Zemao Gu

**Affiliations:** 1 College of Fisheries, Shuangshui Shuanglü Institute, Huazhong Agricultural University, Wuhan 430070, China; 13008330671@163.com (Y.J.); hzauxuzhiwei@webmail.hzau.edu.cn (Z.X.); pmm@webmail.hzau.edu.cn (M.P.); xufama@mail.hzau.edu.cn (X.M.); 2 Hubei Hongshan Laboratory, Wuhan 430070, China; yujiaqian0421@163.com (J.Y.); 19972825718@163.com (Z.F.)

**Keywords:** rice–duck–crayfish integrated model, phytoplankton, zooplankton, community structure, diversity, correlation analysis, environmental factor

## Abstract

To accurately manage precise feeding and water quality regulation in the rice–duck–crayfish integrated system (RDCI), the continuous monitoring of plankton and physicochemical parameters in the water was conducted from March 2022 to January 2023 in both the RDCI and the rice–crayfish continuous culture system (RCCC). The results showed that a total of 188 phytoplankton species and 92 zooplankton species were identified in the RDCI, whereas 152 phytoplankton species and 95 zooplankton species were detected in the RCCC. The phytoplankton community composition was similar between these two systems. For zooplankton, Rotifera was the dominant group. However, Chlorophyta and Bacillariophyta were the dominant phytoplankton groups. Compared with the RCCC, the RDCI exhibited lower plankton density during the crayfish-farming stage and overwintering stage, but higher plankton biomass during the crayfish-farming stage, overwintering stage, and rice maturity stage. The diversity indices, richness indices, and evenness indices of both phytoplankton and zooplankton in the RDCI were significantly higher than those in the RCCC. Correlation analysis indicated that water temperature, dissolved oxygen, total nitrogen, and ammonia nitrogen were the key environmental factors affecting plankton community structure. In summary, compared with the RCCC, the RDCI exhibits higher plankton diversity and better evenness, suggesting a more complex and stable community structure. The species composition of plankton and related indices indicate that the RDCI mitigates the degree of eutrophication in water during both the crayfish farming and the overwintering stages, while increasing nutrients levels during the rice planting stage. This approach is beneficial for reducing non-point-source pollution in agriculture and promoting green agricultural development.

## Simple Summary

To clarify plankton dynamics and improve water quality management in the rice–duck–crayfish integrated system (RDCI), we continuously monitored plankton and physicochemical parameters in the RDCI and the rice–crayfish continuous culture system (RCCC) (Mar 2022–Jan 2023). Key findings include the RDCI had 214 phytoplankton/92 zooplankton species (vs. 196/95 in the RCCC), with shared dominant groups (Chlorophyta, Bacillariophyta; Rotifera). Compared with the RCCC, the RDCI had lower plankton density, higher biomass in key stages, and significantly higher plankton diversity indices. Key influencing factors were water temperature, dissolved oxygen, total nitrogen, and ammonia nitrogen. The RDCI has a more stable plankton community, alleviates eutrophication in crayfish stages, increases rice-stage nutrients, and supports green agriculture.

## 1. Introduction

Red swamp crayfish (*Procambarus clarkii*, *P*. *clarkii*) has become one of the most extensively cultivated freshwater species in China, with its farming area approaching two million hectares [1]. Among various farming approaches, the rice–crayfish continuous culture system (RCCC) is widely adopted for its efficient utilization of the spatiotemporal structure, water resources, and soil fertility of paddy fields. This model establishes a symbiotic relationship between crayfish and rice [2,3]. Benefiting from such synergistic interactions, the RCCC delivers remarkable ecological, economic, and social benefits [4,5,6]. A distinctive advantage of this system lies in its self-contained cycle of “self-breeding, self-rearing, and integrated cultivation”, which has effectively ensured the supply of crayfish seedlings and strongly supported the rapid expansion of the crayfish industry [7]. However, this long-term closed-loop approach has brought unintended consequences: the accumulation of residual crayfish populations, uncontrolled density levels, and escalating weed proliferation [8]. Mechanistically, the closed-loop system’s limited and size-selective harvesting failed to remove juvenile crayfish effectively, allowing them to persist and mature into breeding adults, thereby driving population buildup. Concurrently, the absence of efficient top-down predators and insufficient foraging pressure in the system enabled crayfish densities to rise unchecked. Their intensive burrowing activities then disrupted plant root systems and reduced competitive pressure on weeds, creating favorable conditions for weed establishment and rapid proliferation [2,7]. These issues have led to inefficiencies in feed management, excessive energy and nutrient input during the crayfish culture phase, nutrient depletion during rice cultivation, and increased reliance on herbicides, ultimately impeding the broader goals of sustainable agriculture [9].

The rice–duck–crayfish integrated system (RDCI), a novel eco-circular model, was established by introducing the dusks into the RCCC to optimize the ecological function of paddy fields. Early field investigations indicated that ducks can effectively suppress weeds through trampling and foraging (except during late-stage rice maturation), regulate crayfish populations via predation, and contribute biological fertilizers through manure, thereby enhancing nutrient synchrony in both space and time [10]. However, the overabundance of duck manure, which has an effect similar to excessive feed input in the RCCC, may compromise water quality and pose environmental risks to the aquaculture component [11]. Increasing attention has been paid to tailwater discharge from rice-integrated farming systems, in which nitrogen (N) and phosphorus (P) serve as key indicators of aquatic ecological health [12]. In the RDCI, N and P mainly originate from crayfish and duck excreta, residual feed, rice straw, and fertilizers, with phytoplankton and zooplankton acting as the major biological nutrient assimilators [13]. As fundamental components of the aquatic food web, phytoplankton support zooplankton growth, and zooplankton grazing further regulates nutrient cycling and water clarity [12,13]; thus, the species composition, abundance, and temporal dynamics of plankton communities not only reflect environmental conditions but also sustain the growth of *P*. *clarkii* [14,15]. Previous studies have demonstrated that integrated rice–aquatic animal systems (e.g., rice–fish, rice–crayfish, rice–crab) often increase water N and P concentrations. While these nutrients initially contribute to trophic enrichment, they simultaneously stimulate plankton blooms and enhance overall biodiversity. Under well-managed, balanced nutrient regimes, such increases can promote primary productivity and strengthen ecosystem functioning [16,17]. In particular, the RCCC supports high plankton density, biomass, and diversity, maintaining a stable natural food supply for *P. clarkii*. Notably, although phytoplankton is an important component of the plankton community, it is not the dominant food source for *P. clarkii*. As primary consumers feeding on phytoplankton, zooplankton connect primary producers to *P. clarkii*, and the high zooplankton abundance in the RCCC ensures the growth and population stability of *P. clarkii*. The differential utilization of these two plankton groups by *P. clarkii* affects energy flow and material cycling [18]. Plankton communities are also strongly regulated by environmental factors such as water temperature and dissolved oxygen (DO). Temperature has been identified as the dominant factor controlling seasonal variations in phytoplankton biomass, while plankton diversity correlates significantly with water temperature (WT), salinity, nitrogen forms, pH, and chemical oxygen demand (COD) [19,20]. These results indicate that plankton assemblages in rice-integrated systems respond sensitively and complexly to interactive biological, management, and environmental variables. Considering the co-culture of crayfish and ducks in the RDCI, its plankton community structure and succession may be more dynamic and complicated than single-species integrated systems [21]. Nevertheless, the composition, structure, and interspecific interactions of plankton communities, particularly the relationship between plankton community dynamics and environmental factors, remain poorly understood in the RDCI.

Therefore, a year-round study was conducted by the continuous sampling of plankton communities and water physicochemical parameters including COD, total phosphorus (TP), active phosphorus (PO_4_^3−^-P), total nitrogen (TN), ammonia nitrogen (NH_4_^+^-N), nitrate nitrogen (NO_3_^−^-N), and nitrite nitrogen (NO_2_^−^-N) in both the RDCI and the RCCC in Jianli County, Hubei Province, China. This investigation focused on comparing species composition, dominant taxa, density, biomass, and diversity indices between the two systems. The goal was to elucidate the distinctive patterns of plankton community structure under the RDCI and assess their ecological correlations with water quality factors, providing a scientific foundation for improved feed precision, water regulation, and model optimization in the RDCI.

## 2. Materials and Methods

### 2.1. Study Area

**Figure 1:**
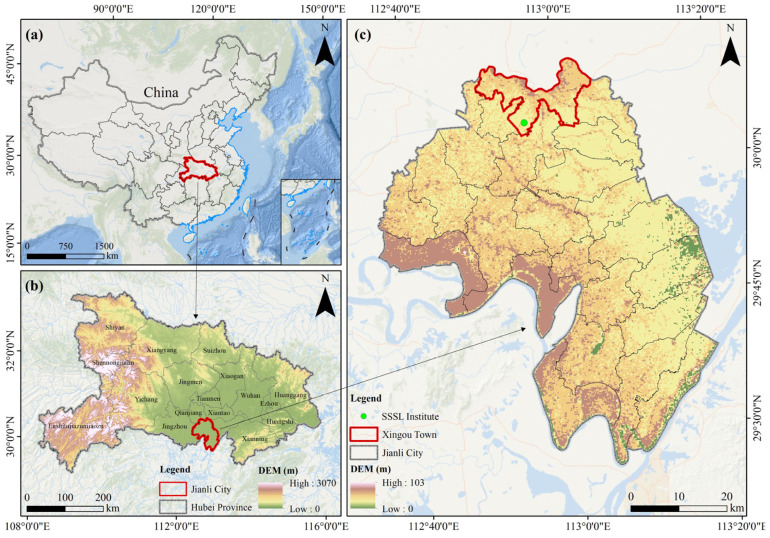
A map of the study. (**a**) Schematic map of the national location of the study site. The province where the study site is located is marked by a red box. (**b**) Schematic map of the provincial location of the study site. The urban area where the study site is located is marked by a red box. (**c**) Schematic map of the precise location of the study site. The town where the study site is located is marked by a red box; Green dots represent the specific experimental sites.

This experiment was carried out at the “Shuangshui Shuanglü Technology Station” in Xingou Town, Jianli County, Hubei Province, China (112°38′ E, 29°59′ N), a typical representative area of rice–aquaculture integrated systems in the central subtropical zone of China, with a mild climate, abundant rainfall, and suitable natural conditions for this type of aquaculture (Figure 1).

### 2.2. Ethics Statement

All animal protocols were approved by the Institutional Animal Care and Use Committee of Huazhong Agricultural University (Approval No. HZAUSW-2018–008).

### 2.3. Experimental Design

Six paddy fields under the RCCC were selected, each covering ~4000 m^2^. Three adjacent fields served as the control group (the RCCC model) and the other three adjacent fields as the experimental group (the RDCI model). The key management differences between the two groups were as follows: the experimental group had duck houses built along outer ridges (connected to fields via machine-accessible walkways) and 100 cm high nylon mesh fences (centered on duck houses) to prevent duck escape. Our previous study demonstrated that Wuqin 10 ducks (bred by Wuhan Academy of Agricultural Sciences) showed significant advantages in weed control, insect removal, and predation on residual *P. clarkii*, making them more suitable for the rice–duck–crayfish integrated farming system (unpublished data). For duck rearing in the experimental group, 20-day-old Wuqin 10 ducks were introduced at 12 ducks/mu (180 ducks/hm^2^) 15 days after rice transplanting, with a 55–60-day rearing period (mid-July to early September). The duck age, stocking density and rearing period were determined based on the rice growth cycle and local optimal farming practices. Such management can effectively control weeds and pests without damaging rice seedlings or affecting rice yield. Ducks were herded into fields daily (6:00–18:00) with one supplementary feeding per day (compound feed (Zhejiang Deqing Hongli Feed Co., Ltd., Ningbo, China) contains corn, soybean meal, wheat middlings, rapeseed meal, wheat bran, amino acids, trace elements, calcium hydrogen phosphate, salt, complex enzyme preparations, and various vitamins required for growth) in the early stage, rice grains in the late stage) at 10% of the current body weight of ducks.

This experiment followed a full production cycle from March 2023 to January 2024, in alignment with the RDCI farming schedule. Sampling was conducted during five critical stages: the crayfish farming stage (March–May), rice reviving stage (June), rice–duck co-culture stage (July–August), rice ripening stage (September), and the overwintering stage (November and January).

Field sampling took place between 8:00 and 10:00 a.m. each day. Water depth was first measured to determine sampling strata. A 2.5 L plexiglass water sampler, pre-rinsed with on-site water, was used to collect samples. To better reflect the overall water environmental characteristics of each plot, the nine-point method was employed, with samples taken from four locations in the surrounding ditches and five within the paddy areas. At each point, samples were collected from the surface (0.1~0.2 m below the surface), mid-water, and near the bottom (0.1~0.2 m above the substrate), yielding a total volume of 20 L/sampling round. For plots with a water depth below 0.3 m, only mid-layer samples were collected. All subsamples collected from each paddy area were homogenized into one single composite sample for subsequent determination, which could effectively represent the overall water quality status and reduce random errors within the plot.

### 2.4. Collection, Identification, and Enumeration of Plankton Communities

Plankton were collected according to the method described by Chen et al. [22]. Both qualitative and quantitative samples of phytoplankton and zooplankton were collected at each sampling site. For qualitative analysis, phytoplankton were captured using a No. 25 plankton net (mesh size: 0.064 mm), while zooplankton were collected with a No. 13 plankton net (mesh size: 0.112 mm). Each net was gently towed horizontally from the surface to a depth of 0.5 m at a velocity of 20~30 cm/s for approximately 1 to 3 min. In sites with water depths exceeding 3 m, sampling was conducted across mixed layers—upper, middle, and bottom strata. After collection, the nets were raised to drain excess water, and the concentrated samples were transferred into 100 mL bottles. Phytoplankton samples were preserved with Lugol’s iodine solution (COOLABER, Beijing, China), while zooplankton samples were fixed using formaldehyde (Servicebio, Wuhan, China) [23,24].

The species-level identification of qualitative samples was performed according to the method described by O’Brien et al. [25]. For quantitative phytoplankton analysis, 1 L of water was collected using a 1 L acrylic hydrophore. Samples were fixed with Lugol’s iodine and left undisturbed for 24 h to allow natural sedimentation. The supernatant was carefully decanted, and the remaining 20~25 mL of concentrated sample was transferred into a 50 mL vial. Following thorough mixing, 0.1 mL was extracted and placed in a 0.1 mL counting chamber for species-level cell enumeration under a microscope (Becton Dickinson, Franklin Lakes, NJ, USA). Protozoa and rotifers were quantitatively counted from the same concentrated phytoplankton samples. Specifically, a 30 mL concentrated phytoplankton sample was used for enumeration; a 0.1 mL volume of this sample was used for protozoan counting and 1 mL was used for rotifer counting. As small protozoa and rotifers can easily pass through the mesh of a No. 13 plankton net, they cannot be effectively collected with net sampling and were therefore quantified from concentrated phytoplankton samples rather than net-collected samples. By comparison, Cladocera and Copepoda were collected by filtering 20 L of water sample through a No. 13 plankton net. All samples were fixed with formaldehyde solution, and Cladocera and Copepoda were enumerated using a 1 mL counting chamber. All quantitative measurements were based on the average of three replicate samples, and results were used to determine the density (individuals (ind)/L) and biomass (mg/L) of the plankton communities.

### 2.5. Detection of Aquatic Environmental Factors

In addition, we determined the physicochemical data for water samples according to the method of Hou et al. [26]. Briefly, COD was measured by the potassium permanganate method; TP was determined using the ammonium molybdate ultraviolet spectrophotometric method; NH_4_^+^-N was analyzed via the Nessler’s reagent ultraviolet spectrophotometric method; NO_3_^−^-N was detected by the phenol disulfonic acid spectrophotometric method; and NO_2_^−^-N was determined using the N-(1-naphthyl)-ethylenediamine spectrophotometric method.

### 2.6. Statistical Analysis

Phytoplankton density (N, ind/L) and biomass (mg/L) were calculated according to the method described by Zhang et al. [27].

(1) $$N=\frac{C}{F}\times \frac{V}{v}\times P$$

where N is the number of phytoplankton cells per liter of water (cells/L); C is the area of the counting chamber (mm^2^); F is the number of microscopic fields observed; V is the concentrated volume of one liter of water sample; v is the volume of the counting chamber (mL); P is the number of counted phytoplankton cells. On the basis of counting, the biomass of plankton is determined by sampling and measuring individual volume, and converting it to mass according to the specific gravity of water.

The dominant plankton species (Y > 0.02) were analyzed according to the method described by Bian et al. [28].

(2) $$Y=n_{i}/N\times f_{i}$$

where Y is the plankton dominance index; ni is the total number of individuals of the ith species; fi is the rate of occurrence of the ith species; and N is the total number of individuals.

To assess plankton community diversity, three widely used alpha diversity indices were computed based on plankton density data: the Shannon–Wiener diversity index (H′), the Margalef richness index (d), and the Pielou evenness index (J′).

(3) $$H^{\prime }=-\sum \left[\left(\frac{ni}{N}\right)\times \ln\left(\frac{ni}{N}\right)\right]$$

(4) d=(S−1)/ln⁡N

(5) $$J\prime =\frac{H^{\prime }}{H\;max}$$

where Pi is the proportion of individuals of a given species in the community; ni is the density of species i obtained during a survey; Fi is the occurrence frequency of a given species across all collected samples; N is the total density of species obtained during a survey; and S is the total number of species obtained during a survey.

All statistical analyses and visualizations were carried out using Microsoft Excel 2019 (Microsoft Corporation, Redmond, WA, USA) and R software version 4.1.3 (R Foundation for Statistical Computing, Vienna, Austria), with the assistance of the R packages vegan, tidyverse, and ggplot2.

## 3. Results

### 3.1. Community Structure Characteristics of Phytoplankton

**Figure 2:**
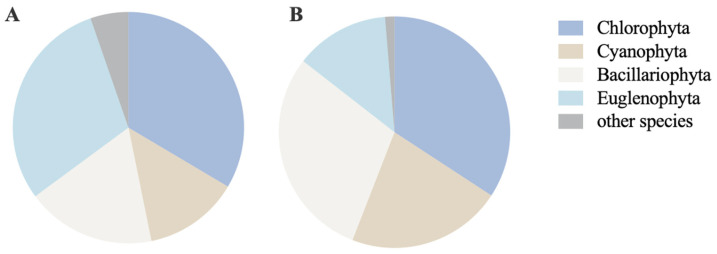
Phytoplankton species composition in RDCI (**A**) and RCCC (**B**).

A total of 188 phytoplankton species belonging to 70 genera and 8 phyla, including Cyanophyta, Chlorophyta, Bacillariophyta, Euglenophyta, Pyrrophyta, Cryptophyta, Chrysophyta, and Xanthophyta, were identified in the RDCI. Based on the species composition of phytoplankton in the RDCI during this experiment (Figure 2A), Chlorophyta, Euglenophyta, and Bacillariophyta were the dominant phyla in paddy water, accounting for approximately 80% of the total phytoplankton species. Specifically, Chlorophyta comprised 63 species in 16 genera (33.51%), Euglenophyta included 56 species in 10 genera (29.79%), and Bacillariophyta consisted of 34 species in 15 genera (18.09%). In the RCCC, 152 phytoplankton species distributed across 62 genera and 8 phyla were detected, and the dominant phytoplankton phyla were consistent with those in the RDCI (Figure 2B).

A total of 19 dominant species (Y ≥ 0.02) were observed in the RDCI, more than the 12 dominant species (Y ≥ 0.02) in the RCCC (Table 1). *Microcystis flos-aquae* was the absolute dominant species in both systems, and dominated in all stages throughout the year (Y > 0.5). Its annual average dominance index was 0.75 in the RDCI, lower than 0.79 in the RCCC. Except for *Microcystis flos-aquae*, the dominant species in the RDCI showed obvious succession characteristics in spatial and temporal distribution. Specifically, nine dominant species (mainly Chlorophyta and Bacillariophyta) were found during the *P. clarkii* culture stage; five dominant species (mainly Cyanophyta and Chlorophyta) occurred during the rice regreening stage; five dominant species (mainly Euglenophyta and Cyanophyta) appeared during the rice–duck co-culture stage; and eight dominant species (mainly Chlorophyta and Bacillariophyta) were present in the rice maturity stage and overwintering stage, with similar community composition. In contrast, the number of dominant species in each stage of the RCCC was lower than that in the RDCI, and no obvious succession of dominant species was observed among different stages. Cyanophyta and Chlorophyta were the main dominant groups, with a small number of Bacillariophyta and Euglenophyta only appearing during the crayfish culture stage.

**Figure 3:**
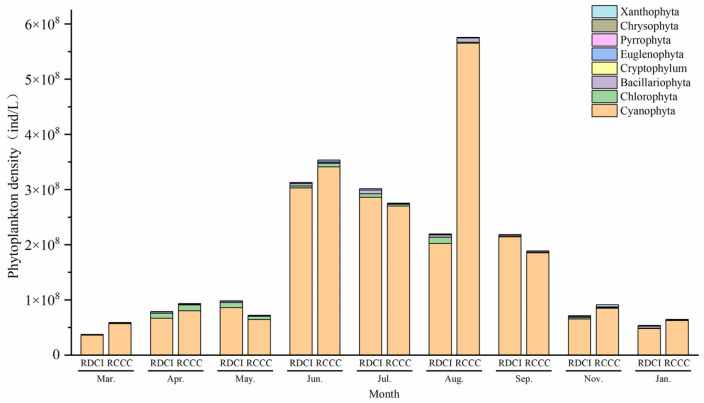
Changes in phytoplankton density in RDCI and RCCC at different stages.

The annual phytoplankton density in the RDCI ranged from 3.75 × 10^7^~3.13 × 10^8^ ind/L, following a characteristic “W-shaped” temporal pattern. Density levels peaked during the crayfish farming stage and again in June (rice reviving stage), followed by a marked decline during the rice–duck co-culture stage. A secondary increase was observed during the rice ripening stage, before dropping sharply to the lowest levels in the overwintering stage. In the RCCC, phytoplankton density exhibited a narrower range, i.e., 5.90 × 10^7^~5.75 × 10^8^ ind/L, with the highest and lowest values recorded in August and January, respectively. Across both systems, Cyanophyta consistently dominated the phytoplankton community during the entire sampling period, accounting for 94.28% of the average total density across all sampling stages in the RDCI and 91.67% in the RCCC. Notably, the proportion of Cyanophyta was lower in the RDCI than in the RCCC during the crayfish farming stage and in January (late overwintering), but exceeded the RCCC levels during all other stages. In contrast, Chlorophyta and Bacillariophyta showed an inverse pattern, contributing more to total density in the RDCI during the crayfish farming and overwintering stages (Figure 3).

**Figure 4:**
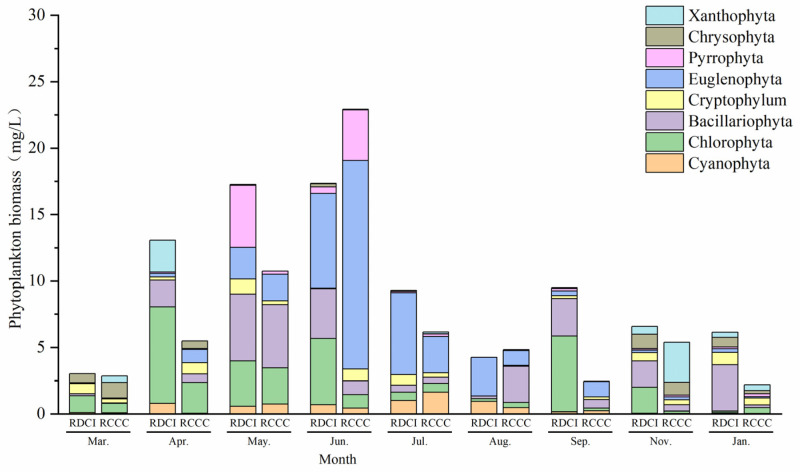
Changes in phytoplankton biomass in RDCI and RCCC at different stages.

The annual average phytoplankton biomass reached 9.61 ± 7.72 mg/L in the RDCI region and 7.00 ± 3.21 mg/L in the RCCC region (Figure 4). Temporally, the RDCI displayed a “W-shaped” fluctuation pattern, consistent with the trend observed in phytoplankton density. Biomass peaked in May at 17.35 ± 4.31 mg/L and dropped to its lowest value of 4.26 ± 1.01 mg/L in August. In contrast, phytoplankton biomass in the RCCC region followed a unimodal trajectory, increasing steadily to a peak of 22.92 ± 7.98 mg/L in June before declining to a minimum of 2.19 ± 0.71 mg/L in January. A comparative analysis across growth stages revealed that the RDCI exhibited higher phytoplankton biomass than the RCCC during the crayfish farming stage, rice ripening stage, and overwintering stage. However, during the rice reviving stage and the rice–duck co-culture phase, the biomass levels in the RDCI were significantly lower than those in the RCCC. In terms of phytoplankton biomass composition, Chlorophyta dominated the RDCI region during both the crayfish farming stage and the rice reviving stage, contributing to over 50% of the total biomass. During the rice–duck co-culture stage, Chlorophyta and Bacillariophyta were co-dominant. The rice ripening stage saw a shift in dominance to Cryptophyta, followed by Euglenophyta in the early overwintering stage and Dinophyta together with Bacillariophyta in the late overwintering stage. In the RCCC region, Chlorophyta similarly led during the crayfish farming and rice reviving stages. However, Euglenophyta became the predominant group throughout all subsequent stages.

In terms of taxon-specific biomass, the annual average levels of Chlorophyta (2.48 ± 0.41 mg/L), Bacillariophyta (2.22 ± 0.78 mg/L), and Cryptophyta (0.53 ± 0.11 mg/L) were higher in the RDCI region compared to the RCCC region. In contrast, Euglenophyta, Cyanophyta, and Xanthophyta exhibited lower average biomass in RDCI, with values of 2.19 ± 0.58 mg/L, 2.49 ± 0.62 mg/L, and 0.38 ± 0.14 mg/L, respectively. Temporal comparisons revealed that Chlorophyta biomass in the RDCI was lower than in the RCCC only during the rice–duck co-culture stage, while it exceeded the RCCC levels in all other stages. Cyanophyta biomass in the RDCI surpassed that in the RCCC only during the rice ripening stage and remained lower throughout the rest of the year. Similarly, Euglenophyta biomass was higher in the RDCI during early overwintering but was consistently lower in all other stages. Notably, Bacillariophyta biomass in the RDCI remained higher than in the RCCC across the entire overwintering stage.

**Figure 5:**
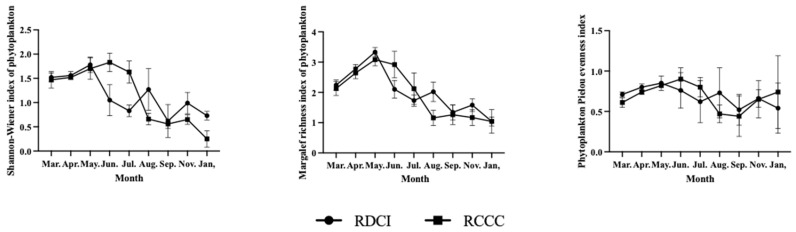
Changes in phytoplankton diversity index in RDCI and RCCC at different stages. Error bars represent standard deviation (SD).

The results of annual variations in phytoplankton diversity indices indicate that the mean values of the d and J’ in the RDCI region are 2.45 ± 0.37 and 0.67 ± 0.11, respectively, both higher than those recorded in the RCCC region (2.34 ± 0.45 and 0.63 ± 0.13). The annual mean of the H′ in the RDCI was 1.15 ± 0.12, nearly identical to the value of 1.16 ± 0.14 observed in the RCCC. Across the year, the three indices in the RDCI exhibited a consistent temporal pattern: increasing during the crayfish farming stage and rice reviving stage, declining during the rice–duck co-culture stage, rising again in the rice ripening stage, and decreasing in the overwintering stage. Notably, all three indices peaked in June, with maximum values of 1.98 ± 0.34 (H′), 3.43 ± 0.38 (d), and 0.88 ± 0.12 (J’), respectively. The lowest H′ and J′ were observed in August (0.55 ± 0.13 and 0.44 ± 0.10), while the d reached its minimum in January (1.72 ± 0.18). In the RCCC region, all three indices followed a unimodal trend—rising during the first half of the year, peaking in June (1.86 ± 0.22, 3.10 ± 0.17, and 0.85 ± 0.11), and subsequently declining to their lowest levels in January (0.52 ± 0.12, 1.08 ± 0.14, and 0.41 ± 0.10). When compared stage by stage, the RDCI region exhibited higher diversity values than the RCCC during the crayfish farming stage, rice reviving stage, rice ripening stage, and overwintering stage. In contrast, during the rice–duck co-culture phase, all three indices in the RDCI dropped below those in the RCCC (Figure 5).

### 3.2. Community Structure Characteristics of Zooplankton

**Figure 6:**
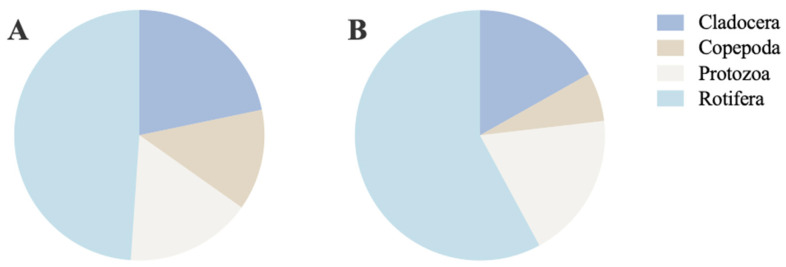
Zooplankton species composition in RDCI (**A**) and RCCC (**B**).

A total of 92 zooplankton species, classified into four major taxonomic groups, were recorded in the RDCI region, compared to 95 species identified in the RCCC region (Figure 6). In the RDCI, the zooplankton community comprised 45 rotifers (48.91%), 20 cladocerans (21.74%), 15 protozoans (16.30%), and 12 copepods (13.04%). By contrast, the RCCC community included 55 rotifers (57.89%), 18 protozoans (18.95%), 16 cladocerans (16.84%), and 6 copepods (6.32%).

A total of 22 dominant zooplankton species were identified in the RDCI region, exceeding the 15 species recorded in the RCCC. The dominant species composition showed considerable overlap between the two systems (Table 2). However, several species were exclusively dominant in the RDCI, including *Bosmina longirostris*, *Simocephalus vetulus*, *Sinocalanus tenellus*, *Lecane papuana*, *Eucyclops serrulatus*, and *Arcella vulgaris*. In contrast, no endemic dominant species were observed in the RCCC region. The dominant species in the RDCI exhibited clear successional shifts in spatiotemporal distribution. Copepods prevailed from March to May, followed by rotifers as the dominant group during June to August, and cladocerans taking over from November to January of the following year. Notably, nauplii maintained dominance throughout the year in both systems. In the RCCC region, rotifers dominated across all seasons, with *Keratella cochlearis* and *Brachionus urceus* consistently identified as the most prominent species.

**Figure 7:**
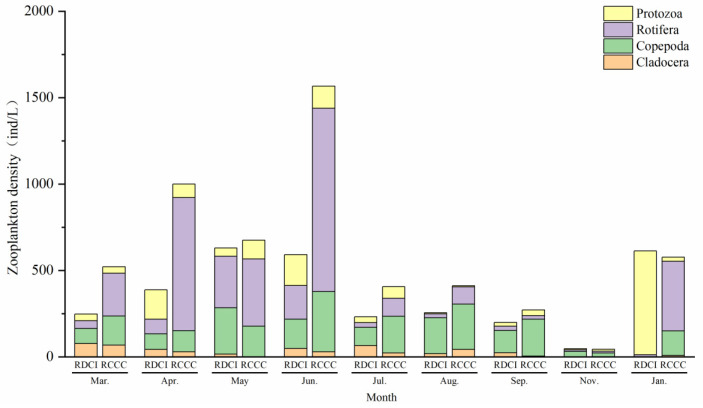
Zooplankton density changes in RDCI and RCCC at different stages.

In addition, in the RDCI region, annual density ranged from 147.00 to 1060.10 ind/L, following a fluctuation pattern characterized by an initial increase, a subsequent decline, and a secondary rise. In contrast, zooplankton density in the RCCC region varied between 274.91 and 2141.47 ind/L, with the highest value observed in March and the lowest in July. Rotifers remained the dominant zooplankton group throughout the year in both systems, contributing to 48.91% and 57.89% of the total density in the RDCI and RCCC regions, respectively (Figure 7).

**Figure 8:**
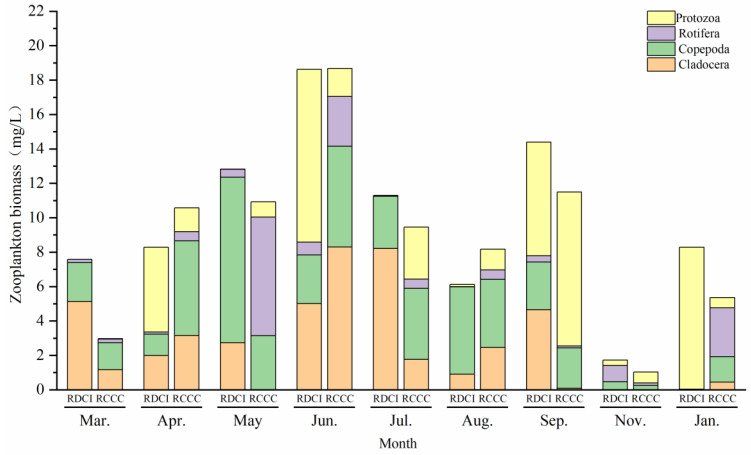
Zooplankton biomass changes in RDCI and RCCC at different stages.

The results of the seasonal variation in zooplankton biomass indicate that the annual average biomass was 12.28 ± 9.77 mg/L in the RDCI region and 8.21 ± 4.29 mg/L in the RCCC region. In terms of temporal dynamics, both systems exhibited a similar trend of increase–decrease–increase. In the RDCI region, zooplankton biomass peaked in September (32.03 ± 6.32 mg/L) and reached its lowest level in August (1.87 ± 0.76 mg/L). In the RCCC region, the maximum and minimum values occurred in September and January, i.e., 17.31 ± 4.17 and 3.18 ± 1.64 mg/L, respectively. Cross-stage comparisons revealed that zooplankton biomass in the RDCI was consistently higher than in the RCCC during the crayfish farming stage, rice reviving stage, and rice ripening stage. However, it was markedly lower during the overwintering and rice–duck co-culture stages. Regarding species composition, protozoans and copepods were the dominant contributors to biomass in the RDCI, together accounting for over 50% of the total zooplankton biomass (Figure 8).

**Figure 9:**
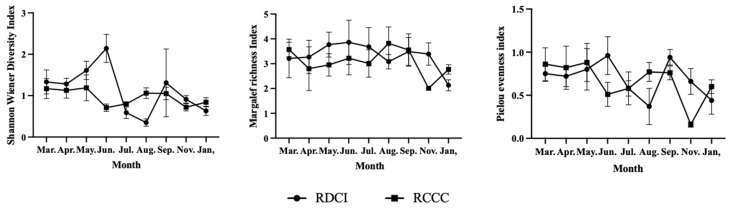
Zooplankton diversity index in RDCI and RCCC at different stages. Error bars represent standard deviation (SD).

We further examined the annual variations in zooplankton diversity indices. Results showed that the mean annual values of the H′ (1.13 ± 0.25), d (3.32 ± 0.57), and J′ (0.69 ± 0.13) were all higher in the RDCI region than in the RCCC region, where the corresponding values were 0.96 ± 0.15, 3.08 ± 0.49, and 0.66 ± 0.13. In the RDCI, all three indices followed a similar seasonal trajectory: increasing during the crayfish farming and rice reviving stages, declining during the rice–duck co-culture stage, rebounding in the rice ripening stage, and falling again during overwintering. Peak values for the H′, d, and J′ were recorded in June, reaching 2.14 ± 0.34, 3.89 ± 0.89, and 0.96 ± 0.22, respectively. The lowest H′ and J′ values occurred in August (0.35 ± 0.09 and 0.37 ± 0.21), while the d reached its minimum in January (2.13 ± 0.22). When compared stage by stage, the RDCI consistently showed higher diversity index values than the RCCC during the crayfish farming stage, rice reviving stage, rice ripening stage, and overwintering stage. In contrast, all three indices dropped below the RCCC values during the rice–duck co-culture stage (Figure 9).

### 3.3. Correlation Analysis Among Plankton Community

**Figure 10:**
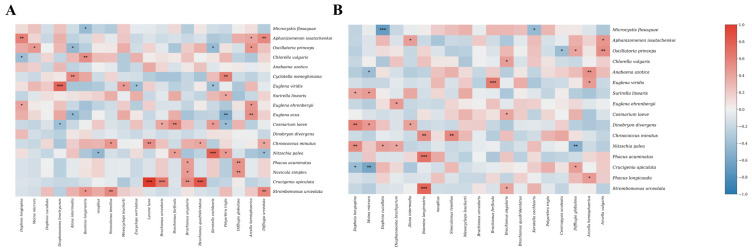
Spearman’s correlations of dominant plankton species in RDCI (**A**) and RCCC (**B**). “*” indicates *p* < 0.05; “**” indicates *p* < 0.01; “***” indicates *p* < 0.001.

Spearman correlation analysis was conducted to assess the associations between dominant zooplankton and phytoplankton species in the two systems. In the RDCI, strong correlations were primarily limited to a few tightly coupled species pairs. By contrast, overall correlations in the RCCC were relatively weak, though several significant positive and negative relationships were still observed. In the RDCI, specific cyanophyte species were significantly positively correlated with cladocerans. For example, *Aphanizomenon issatschenkoi* showed a significant correlation with *Diaphanosoma brachyurum* (r = 0.514), while *Oscillatoria princeps* exhibited a stronger association with *Moina micrura* (r = 0.618). In the RCCC, the bacillariophyte *Nitzschia palea* displayed positive correlations with *Daphnia cucullata* (r = 0.610) and *S. tenellus* (r = 0.633). Similarly, in the RDCI, *N. palea* also showed a strong positive correlation with *Alona intermedia* (r = 0.612). As for negative correlations, beyond *M. flos-aquae*, several bacillariophytes in the RDCI were negatively associated with copepods. Notably, *N. palea* had a significant negative correlation with *S. tenellus* (r = −0.436). In the RCCC, both *D. cucullata* and *Difflugia hemisphaerica* were negatively correlated with *M. flos-aquae*, with coefficients of −0.668 and −0.669, respectively. Additionally, *Mesocyclops leuckarti* was negatively associated with *Chlorella vulgaris* (r = −0.396) (Figure 10).

### 3.4. Correlations Between Plankton and Aquatic Environmental Factors

Spearman correlation analysis was performed to examine the relationships between plankton community structure and aquatic environmental variables in both the RDCI and RCCC. Results identified WT and NH_4_^+^–N as the primary drivers shaping plankton community dynamics. In both systems, WT exhibited a significant positive correlation with phytoplankton density (phy-density) and biomass (phy-biomass), highlighting its key regulatory role. Additionally, NH_4_^+^–N was significantly negatively correlated with the H′ (pz-shannon) in both systems. In the RDCI, nutrient-related variables exerted further inhibitory effects on phytoplankton diversity. TN showed significant negative correlations with both pz-shannon (r = −0.49, *p* = 0.009) and J′ (pz-pielou, r = −0.43, *p* = 0.026). Zooplankton community diversity was influenced primarily by DO and WT. DO was significantly positively correlated with the zooplankton J′ (zo-pielou, r = 0.39, *p* = 0.046), and showed a highly significant positive correlation with the d (zo-margalef, r = 0.52, *p* = 0.005). In contrast, WT was strongly negatively associated with zo-margalef (r = −0.57, *p* = 0.002). Moreover, TN also negatively impacted zooplankton density (zoo-density, r = −0.45, *p* = 0.020).

**Figure 11:**
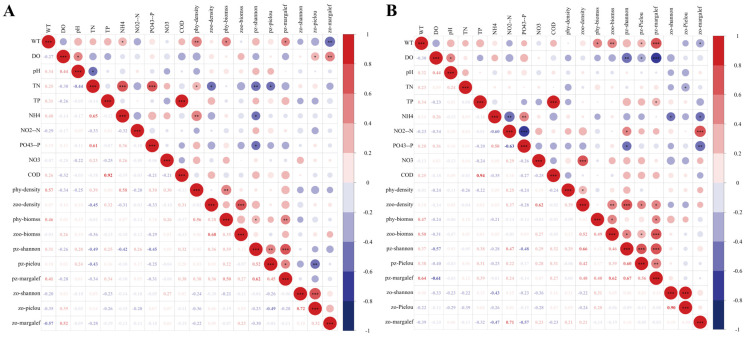
Spearman correlation analysis between biomass, density, biodiversity indices of phytoplankton (“P”) and zooplankton (“Z”), and environmental variables in RDCI (**A**) and RCCC (**B**). “*” indicates *p* < 0.05; “**” indicates *p* < 0.01; “***” indicates *p* < 0.001.

In the RCCC, phytoplankton diversity indices were more sensitive to environmental gradients. Pz-shannon showed a significant positive correlation with nitrite nitrogen (NO_2_^−^–N, r = 0.47, *p* = 0.014), but was negatively correlated with DO (r = −0.57, *p* = 0.002) and phosphate phosphorus (PO_4_^3−^–P, r = −0.48, *p* = 0.011). Pz-margalef exhibited a highly significant positive correlation with WT (r = 0.64, *p* < 0.001) and a significant negative correlation with DO (r = −0.61, *p* = 0.001). Zooplankton structure in the RCCC was particularly responsive to N forms. NO_3_^−^–N was highly positively correlated with zoo-density (r = 0.62, *p* < 0.001), while NO_2_^−^–N exhibited a strong positive association with zo-margalef (r = 0.71, *p* < 0.001). Conversely, TN was significantly negatively correlated with zo-pielou (r = −0.39, *p* = 0.042) (Figure 11).

## 4. Discussion

### 4.1. Rice–Duck–Crayfish Integration Regulates Phytoplankton Community and Improves Ecosystem Stability and Sustainability

In this study, the annual phytoplankton community structure was compared between the RDCI and the conventional RCCC. Regarding the phytoplankton species, its richness in the RDCI was higher than in the RCCC. In addition, its seasonal variation is consistent with that of previous studies on rice–aquaculture integrated systems [29]. It was demonstrated that species detection can be affected by the differences in aquaculture environments, with the duck activity in paddy fields as the main factor causing environmental differences. During the rice–duck co-culture stage, the discrepancies in species richness reached the most significant extent. Specifically, high temperature and unstable nutrient conditions increased species richness in the RCCC [30], while the activity of ducks reduced water transparency, thereby decreasing the abundance of Chlorophyta, which require relatively high light intensity [28,31]. In our study, we observed that, during the rice maturity stage after ducks were removed from the paddy fields, the species number of Chlorophyta in the RDCI recovered, and Chlorophyta regained dominance. It is speculated that light availability is also one of the key regulatory factors affecting the phytoplankton community in this system. As a more even community structure, the RDCI maintained more dominant species. This aligns with the dominant phyla characteristics discovered in previous rice–aquaculture integrated systems [29,32]. Among them, Bacillariophyta gained dominance in cooler seasons, while Cyanophyta and Chlorophyta persisted throughout the year. This result is consistent with previous findings [29]. Notably, the RDCI promoted the growth of beneficial taxa but reduced the dominance of the detrimental cyanobacterium *Microcystis flos-aquae* [33]. Differently, Euglenophyta proliferated significantly only during the turbid rice–duck co-culture stage in RDCI, which illustrates their low light tolerance [34]. Both systems were characterized by high Cyanophyta density and high Chlorophyta biomass, which reaffirm these features as typical community structure traits of rice–aquaculture integrated systems.

Phytoplankton biomass and density exhibited a unique “W-shaped” seasonal pattern in the RDCI, which differs from traditional rice–crayfish co-culture. Also, this resulted mainly from the regulatory effect of duck activity. During the farming of *P*. *clarkii* (crayfish), nutrient input promoted phytoplankton growth [35]. Nevertheless, the effect of light limitation outweighed that of nutrient input during the rice–duck co-culture stage [31]. After duck removal, the explosive growth of Euglenophyta and Cryptophyta was promoted by the organic matter produced by the decomposition of residual feed and duck manure, while the overall growth of phytoplankton was inhibited by low winter temperatures [36]. In addition, duck predation suppressed the natural recruitment of *P*. *clarkii* [37], reduced nutrient loading, lowered the density of *Microcystis flos-aquae*, and increased the proportion of beneficial Chlorophyta [33]. During the rice–duck co-culture stage, the lower phytoplankton biomass in the RDCI facilitated the allocation of nutrients to rice, with a stable rice yield maintained consequently despite a 30% reduction in fertilizer input [38]. Differently, the higher phytoplankton biomass in the RDCI provided critical natural bait for *P*. *clarkii* larvae during winter [39,40,41]. Phytoplankton diversity metrics reflected greater stability in the RDCI during the crayfish farming, rice reviving, ripening, and overwintering stages. These values indicate higher community stability [42]. The seasonal peaks and declines of diversity were found consistent with the changes in Cyanophyta dominance. This is an effect further enhanced in the RDCI by reduced light availability [33].

As revealed by water environment monitoring, the stage of high phytoplankton diversity coincided with the peak discharge stage, which mitigates ecological risks. Relative to natural riverine environments, both aquaculture systems were slightly lower in phytoplankton diversity but higher in evenness. This relates to the anthropogenic pressure from aquaculture activities. Additionally, it exhibited a greater nutrient retention capacity as the concentrations of TN and TP in the tailwater of the RDCI were significantly lower than those in the RCCC [43].

### 4.2. Rice–Duck–Crayfish Integration Enhances Zooplankton Community Stability and Structural Complexity

Regarding taxonomic composition, there were 92 and 95 zooplankton species (across four major groups) identified in the RDCI and RCCC, respectively, both of which were dominated by Rotifera. These counts were slightly lower than those indicated in previous studies [39,44], likely resulting from the variations in sampling stages, species combinations, and environmental heterogeneity. Despite numerical differences, both systems were common in Rotifera predominance with comparable Cladocera and Copepoda proportions [45]. This suggests the shallow water depth and eutrophication potential typical of rice field ecosystems, as demonstrated in prior research.

Our results showed that zooplankton species richness in both systems exhibited a seasonal trend of “increase–decrease–increase”, but these two systems differed in the timing of changes. In the RDCI, richness peaked during the crayfish farming stage, followed by a decline to the lowest during rice–duck co-culture. Ducks can directly prey on zooplankton, especially cladocerans and copepods, thereby reducing their abundance and species number. Meanwhile, duck activities disturb the water column, which affects phytoplankton distribution and further reduces food availability for zooplankton, leading to an obvious decrease in zooplankton species richness (duck predation pressure and disturbance) [46,47]. In the RCCC, richness decreased most significantly during overwintering. A low temperature can inhibit the metabolism, growth and reproduction of zooplankton and increase their mortality. Meanwhile, many zooplankton tend to enter dormancy or diapause to withstand harsh environments, resulting in a significant reduction in species richness [48].

In the RDCI, there were more dominant species supported across all stages, indicating a higher level of ecological niche differentiation and structural complexity. Stage-specific analysis revealed taxonomic shifts in the RDCI. To be specific, Copepoda and Protozoa dominated crayfish farming (crayfish’s selective grazing reshaped interspecific competition), while Rotifera dominated rice–duck co-culture and ripening (duck-induced nutrient elevation fostered Rotifera reproduction), and Cladocera dominated winter (cold resistance via antifreeze proteins). In contrast, Rotifera dominated RCCC year-round [44], facilitated by stable conditions in the absence of ducks, which underscores the ecological resilience of Rotifera in undisturbed environments.

Community structural differences were reflected in the temporal dynamics of zooplankton density and biomass mirrored. Consistent with previous findings [46], density ranking is Rotifera > Protozoa > Copepoda > Cladocera, which demonstrates the dominance of small-bodied taxa in rice fields. Rotifera and Protozoa densities increased notably during rice–duck co-culture in both systems [44,47], linked to enhanced nutrients. Also, the RDCI was significantly higher in Rotifera density [47]. Mainly driven by Copepoda and Cladocera (higher individual dry mass), biomass complemented density trends to reflect resource allocation.

In both systems, zooplankton biomass peaked during rice ripening (optimal temperature and food from organic decomposition) and declined sharply during overwintering (low temperature-induced metabolic suppression). Notably, Cladocera contributed 45.6% to the RDCI winter biomass, associated with the rice straw decomposition-derived food sources within their filter-feeding range [48].

The RDCI had significantly higher values than the RCCC, as confirmed by zooplankton diversity indices. This illustrates lower nutrient concentrations and more balanced species distribution, which is consistent with the ecological principle that eutrophication simplifies communities and reduces resilience. Supported by favorable vs. adverse environmental conditions, seasonal trends (peak in ripening, nadir in overwintering) were accompanied by the variations in biomass and richness [49,50].

### 4.3. Dominant Phytoplankton and Zooplankton Species

In the RDCI, the cyanophyte species *M. flos-aquae* exhibited significant negative correlations with *B. longirostris* (Cladocera), *S. tenellus* (Copepoda), and *D. hemisphaerica* (Protozoa). This could be attributed to ducks directly preying on Cladocera and other small zooplankton, thus reducing their abundance and weakening top-down grazing pressure on phytoplankton. Meanwhile, *M. flos-aquae* can release microcystins that inhibit feeding, survival, and reproduction of zooplankton, further suppressing zooplankton populations. In addition, the physical disturbance generated by duck foraging and movement reduces water transparency and light availability, which favors the competitive dominance of *M. flos-aquae* over other phytoplankton such as Chlorophyta, and the tough cell wall of *M. flos-aquae* lowers its digestibility by zooplankton, making it less vulnerable to grazing [16,51]. Therefore, the negative correlation between *M. flos-aquae* and zooplankton does not reflect a simple unidirectional effect. Instead, ducks mainly act by reducing zooplankton via predation and increasing the competitive advantage of *M. flos-aquae* via disturbance, which together lead to the strong negative correlation observed in the RDCI.

In contrast, several strong positive correlations were identified, including those between *A. issatschenkoi* (Cyanophyta) and *D. brachyurum* (Cladocera), *O. princeps* (Cyanophyta) and *Moina micrura* (Cladocera), *C. vulgaris* (Chlorophyta) and Rotifera, and *N. palea* (Bacillariophyta) and *A. intermedia* (Cladocera). These relationships align with the “high-quality prey promotes species synergy” hypothesis [52]. As non-toxic, nutrient-rich algae, these taxa benefit from elevated N and P levels supplied by duck manure, which promotes algal proliferation. Meanwhile, *P. clarkii* exerts moderate predation on zooplankton, facilitating a relatively stable “algae–zooplankton” food web. Particularly noteworthy is the strong correlation observed between *Strombomonas jarrica* (Euglenophyta) and *Brachionus angularis* (Rotifera), which has rarely been documented in rice–duck–crayfish systems. However, it is comparable to the species-specific algae–Rotifera associations reported by Song et al. [53] in constructed wetland environments, likely reflecting niche co-adaptation under the combined influence of duck-derived nutrients and moderate crayfish disturbance.

The correlation patterns in the RCCC shared both similarities and notable differences with those in the RDCI. Similar to RDCI, positive associations were observed between *N. palea* and both *D. cucullata* (Cladocera) and *S. tenellus* (Copepoda), supporting Chae et al. [54] that Bacillariophyta serve as primary zooplankton prey in rice–crayfish systems. In the RCCC, benthic disturbance by *P. clarkii* enhanced light penetration, promoting Bacillariophyta growth and thereby supporting zooplankton development due to their high nutritional quality.

However, two key differences emerged. Firstly, the negative correlation coefficients between *D. cucullata*, *D. hemisphaerica*, and *M. flos-aquae* were more pronounced in the RCCC than in the RDCI, supporting Kudela’s [55] assertion that harmful algal suppression is more significant under single-species disturbance. In the absence of ducks, the RCCC relies solely on *P. clarkii*, whose limited algal grazing capacity allows *M. flos-aquae* to aggregate and release toxins that more effectively inhibit zooplankton. Secondly, the positive correlations between *Dinobryon divergens* (Chrysophyta) and *Brachionus forficula* (Rotifera), and between *S. jarrica* and *Polyarthra trigla* (Rotifera), were significantly stronger than previously reported by Yang et al. [56]. This may be attributed to the lower crayfish stocking density in this study, which reduced predation pressure and helped stabilize the algae–zooplankton trophic relationship. Additionally, the observed positive associations between *Phacus acuminatus* (Euglenophyta) and *P. trigla* or *E. serrulatus* (Copepoda) in the RCCC are relatively rare. These patterns may stem from the site-specific environmental conditions, where optimal WT (25~28 °C) and TN:TP ratios (~15:1) favor *P. acuminatus* growth. Its nutrient profile appears well suited to the dietary preferences of the associated zooplankton, highlighting the role of regional environmental heterogeneity in shaping planktonic interactions.

The fundamental distinction between the RDCI and the RCCC lies in the synergistic influence of biological disturbance and nutrient cycling. In the RDCI, the combined effects of duck activity and *P. clarkii* disturbance created a more homogenized water column with elevated DO levels. Duck manure supplied concentrated nutrients, promoting dominance by key algal species such as *M. flos-aquae* and *C. vulgaris*. These dominant algae exerted more direct effects on zooplankton, facilitating the emergence of strongly coupled interspecific relationships. In contrast, the RCCC’s reliance on single-species (*P. clarkii*) disturbance reduced algal dominance and introduced variability via sediment resuspension, which, combined with selective feeding on small-bodied Cladocera, filtered out less tolerant zooplankton species and diversified interaction patterns.

Furthermore, nutrient form played a decisive role. In RDCI, NH_4_^+^–N derived from duck manure favored *M. flos-aquae* proliferation, exacerbating its inhibitory impact on zooplankton. Conversely, the RCCC generated a more diverse N profile, particularly with elevated NO_2_^−^–N concentrations from crayfish excretion and residual bait decomposition. This N composition favored Bacillariophyta development, reinforcing positive trophic links. These findings support the conceptual model proposed by Yan et al. [57], which posits that “biological disturbance type determines nutrient form, which in turn modulates plankton interaction patterns”.

### 4.4. Aquatic Environmental Factors Regulate Plankton Community Dynamics in RDCI and RCCC

Plankton communities are highly sensitive to environmental fluctuations, and their composition can be readily influenced by both internal and external factors. Internal drivers primarily include species turnover and interspecific competition, while external influences stem from environmental parameters such as N and P cycling. Phytoplankton, in particular, respond rapidly to changes in water quality, with their species composition and abundance closely tied to nutrient dynamics. Li et al. [58] demonstrated that N and P synergistically promote phytoplankton proliferation. Given their position at the base of the aquatic food web, phytoplankton abundance indirectly regulates zooplankton population dynamics through trophic interactions.

Pearson correlation analysis in this study identified WT, TN, and NH_4_^+^–N as the primary environmental variables influencing phytoplankton density and biomass in the RDCI. WT was the dominant factor regulating phytoplankton cell abundance in rice field ecosystems, with higher temperatures (within a certain range) enhancing photosynthetic activity and promoting rapid growth and reproduction. As essential macronutrients, N and P directly influence both the quantity and structure of phytoplankton communities. The RDCI exhibited relatively elevated N and P concentrations. During the crayfish farming stage, nutrient enrichment primarily originated from feed input and fecal discharge by *P. clarkii*. Spring temperatures (25~27 °C) provided optimal conditions for phytoplankton growth. However, after ducks were introduced, their bioturbation increased TN and TP concentrations while simultaneously reducing water transparency. This led to a sharp decline in Chlorophyta, the primary contributors to phytoplankton biomass. Following the rice–duck co-culture stage, phytoplankton biomass in the RDCI rebounded, with Euglenophyta and Cryptophyta showing significant proliferation. This can be attributed to (1) their adaptation to low-light environments during the rice ripening stage and (2) sustained nutrient release from the microbial decomposition of residual duck manure. Both Euglenophyta and Cryptophyta thrive in environments rich in dissolved organic matter.

In the RCCC, WT was significantly positively correlated with phytoplankton biomass and the d. In the absence of duck-induced disturbance, phytoplankton growth followed a more seasonally driven trajectory. Notably, species richness peaked in August, which coincided with the rice–duck co-culture stage in the RDCI. Elevated WT (27.32 °C) during this period favored rapid algal reproduction.

Regarding zooplankton, WT and DO were identified as the principal drivers of community structure and diversity in the RDCI. Yang et al. [59] reported DO, chlorophyll-a, and pH as the major determinants of zooplankton composition in Dianchi Lake, while Pu et al. [53] highlighted the roles of WT, NH_4_^+^–N, and pH in Lianhuan Lake. These findings suggest that geographic location, hydrology, habitat characteristics, and anthropogenic disturbances collectively influence which environmental factors most significantly affect zooplankton dynamics. Li et al. [60] further emphasized that key factors such as NH_4_^+^–N, TN, chlorophyll-a, and water transparency can vary in importance within the same water body across different time stages.

In the RDCI, DO showed significant or highly significant positive correlations with the J′ and d, indicating that elevated DO levels promote species coexistence and a balanced community structure. In contrast, WT exhibited a significant negative correlation with the d. During the rice–duck co-culture stage, WT peaked at 31.96 °C, and concurrent duck tilling led to a marked reduction in Chlorophyta biomass, which is the main food source of zooplankton, thus contributing to a decline in zooplankton diversity.

In the RCCC, NH_4_^+^–N, NO_2_^−^–N, and TN emerged as more dominant regulatory factors for the zooplankton community. NH_4_^+^–N was significantly negatively correlated with both the H′ and d, while TN was significantly negatively correlated with the J′. These results suggest that elevated NH_4_^+^–N and TN concentrations degrade habitat quality, primarily through ammonia toxicity and eutrophication, leading to reduced zooplankton species richness and a more uneven community structure.

## 5. Conclusions

This study systematically revealed the basic characteristics and ecological functions of plankton communities in the RDCI through the continuous monitoring and comparative analysis of plankton and water physicochemical parameters, providing effective technical support for precise feeding management and water quality regulation in integrated rice–aquatic animal culture systems. The study findings not only confirm the typical community characteristics of plankton in rice–crayfish-based culture systems (with Chlorophyta, Bacillariophyta, and Rotifera as dominant groups) but also reveal the unique ecological advantages of the RDCI compared to the RCCC, including higher plankton diversity, more stable community structure, and better water quality regulation capacity. These results lay an important foundation for subsequent in-depth research into the interaction mechanism between plankton communities and the RDCI and provide valuable theoretical resources for the optimization of integrated rice–duck–crayfish culture modes.

## Figures and Tables

**Table 1:** Common species and relative abundance of phytoplankton in RDCI and RCCC from March 2022 to January 2023.

Species		RDCI									RCCC								
		Mar.	Apr.	May	Jun.	Jul.	Aug.	Sep.	Nov.	Jan.	Mar.	Apr.	May	Jun.	Jul.	Aug.	Sep.	Nov.	Jan.
Cyanophyta	*Microcystis flos-aquae*	0.88	0.58	0.78	0.83	0.84	0.88	0.74	0.89	0.90	0.82	0.82	0.84	0.03	0.83	0.85	0.81	0.91	0.89
	*Oscillatoria princes*	-	0.03	0.02	0.07	0.07	-	-	-	-	0.04	0.02	-	0.02	0.04	0.02	-	-	-
	*Aphanizomenon issatschenkoi*	-	-	-	0.03	0.02	0.02	-	-	-	-	-	-	0.06	0.03	0.03	0.05	-	-
	*Anabaena azotica*	-	-	-	-	-	-	-	-	-	-	-	0.02	-	-	0.05	-	-	-
	*Chroococcus minutus*	-	0.15	-	0.02	-	-	-	-	0.02	-	0.02	0.03	0.04	0.03	0.02	0.06	-	-
Chlorophyta	*Chlorella vulgaris*	0.05	0.17	0.80	0.04	-	-	0.06	0.02	-	0.12	0.06	0.04	0.02	-	-	0.06	-	-
	*Cosmarium laeve*	-	-	-	0.02	-	-	0.05	-	-	-	-	-	-	-	-	-	-	-
	*Crucigenia apiculata*	-	-	-	-	-	-	0.06	-	-	-	-	0.02	0.03	-	0.03	-	-	-
Bacillariophyta	*Navicula simplex*	-	-	-	0.02	-	-	0.03	-	-	-	-	0.04	-	-	-	-	-	-
	*Surirella linearis*	0.05	-	-	-	-	-		0.03	0.02	-	-	-	-	-	-	-	-	-
	*Cyclotella meneghiniana*		0.02	0.06	-	-	-	0.02			-	-	-	-	-	-	-	-	-
	*Nitzschia palea*		0.02		-	-	-	0.02	0.03	0.02	-	0.04	-	-	-	-	-	0.04	0.05
	*Synedra ulna*	-	-	0.02	-	-	-	-	-	-	-	0.04	-	-	-	-	-	0.02	0.03
	*Synedra acus*	-	-	0.02	-	-	-	-	-	0.02	-	-	-	-	-	-	-	-	-
Euglenophyta	*Strombomonas urceolata*	0.02	-	-	-	-	-	-	-	-	-	-	-	-	-	-	-	-	-
	*Phacus acuminatus*	-	-	-	-	-	0.03	-	-	-	-	-	-	-	-	-	-	-	-
	*Euglena acus*	-	0.04	0.02	-	-	-	-	-	-	0.02	-	-	-	-	-	-	-	-
	*Euglena ehrenbergii*	-	-	-	-	0.04	-	-	-	-	-	-	-	-	-	-	-	-	-
	*Euglena viridis*	-	-	-	-	0.02	0.03	0.02	-	-	-	-	-	-	-	-	-	-	-
Chrysophyta	*Dinobryon divergens*	-	-	-	-	0.02	0.03	-	-	-	-	-	-	-	0.02	-	-	0.04	-

**Table 2:** Common species and relative abundance of zooplankton in RDCI and RCCC from March 2022 to January 2023.

Species		RDCI									RCCC								
		Mar.	Apr.	May	Jun.	Jul.	Aug.	Sep.	Nov.	Jan.	Mar.	Apr.	May	Jun.	Jul.	Aug.	Sep.	Nov.	Jan.
Protozoa	*Difflugia globulosa*	-	0.24	0.05	0.16	-	-	-	0.12	0.23	-	-	0.23	-	-	-	0.02	0.03	-
	*Arcella hemisphaerica*	-	-	-	-	-	-	-	-	0.12	-	-	-	-	0.02	-	-	-	0.03
	*Arcella vulgaris*	-	-	-	-	0.12	-	0.05	-	0.17	-	-	-	-	-	-	-	-	-
	*Leprotintinnus fluviatile*	-	-	0.13	-	-	-	-	-	0.13	-	-	-	-	-	-	0.03	-	0.02
Rotifera	*Lecane papuana*	-	-	-	-	-	-	-	-	-	-	-	-	-	-	0.09	0.03	0.09	0.08
	*Brachionus urceus*	-	0.22	0.16	0.05	0.25	-	-	-	-	0.04	-	0.03	0.23	0.32	0.12	0.32	-	0.12
	*Brachionus forficula*	-	-	-	-	-	-	0.25	-	-	-		0.11	-	-	0.10	0.22	0.02	-
	*Brachionus angularis*	-	-	0.17	0.03	0.05	-	-	-	-	-	0.08	0.29	0.12	0.22	0.08	-	0.09	0.08
	*Brachionus calyciflorus*	-	-	-	0.17	-	-	-	0.05	-	-	0.16	-	0.08	-	-	-	-	-
	*Keratella cochlearis*	0.08	0.15	0.08	-	0.33	-	-	-	-	0.08	-	0.03	0.12	0.09	0.05	0.05	-	-
	*Polyarthra trigla*	0.04	-	-	-	-	0.03	-	-	-		-	-	0.16	0.05	0.04	0.09	0.04	-
Cladocera	*Diaphanosoma leuchtenbergianum*	-	-	0.08	-	-	-	-	0.09	-	-	-	-	-	-	-	-	0.09	0.13
	*Daphnia cucullata*	0.22	0.06	-	-	-	-	0.02	-	0.12	-	-	-	-	-	-	-	0.10	0.09
	*Diaphanosoma brachyurum*	0.02	-	-	-	-	-	-	-	-	-	-	-	-	-	-	-	-	-
	*Alona intermedia*	0.41	0.03	-	-	-	0.13	-	-	0.08	017	-	-	-	-	-	-	-	-
	*Bosmina longirostris*	0.12	0.03	-	-	-	-	-	-	-	0.03	-	-	-	-	-	-	-	-
	*Simocephalus vetulus*	0.05	-	-	-	0.10	0.11	-	-	0.10	0.10	0.09	-	-	-	-	-	0.09	0.02
Copepoda	*Sinocalanus tenellus*	-	-	0.03	-	-	-	-	-	-	0.03	-	-	-	0.11	0.09	0.02	0.02	-
	*Mesocyclops leuckarti*	0.04	0.03	0.16	-	0.10	0.23	0.25	0.24	-	0.26	0.35	0.10	0.08	0.08	0.13	-	0.10	0.02
	*Eucyclops serrulatus serrulatus*	-	-	-	0.09	-	0.13	-	-	-	-	-	-	-	-	-	-	-	-
	Nauplii	0.02	0.24	0.14	0.50	0.05	0.37	0.43	0.45	0.05	0.29	0.32	0.21	0.27	0.11	0.30	0.22	0.33	0.41

## Data Availability

The original contributions presented in this study are included in the article. Further inquiries can be directed to the corresponding authors.
